# Poly(ethylene glycol) brush-*b*-poly(*N*-vinylpyrrolidone)-based double hydrophilic block copolymer particles crosslinked *via* crystalline α-cyclodextrin domains†

**DOI:** 10.1039/c8ra10672j

**Published:** 2019-02-08

**Authors:** Noah Al Nakeeb, Zdravko Kochovski, Tingting Li, Youjia Zhang, Yan Lu, Bernhard V. K. J. Schmidt

**Affiliations:** Max-Planck Institute of Colloids and Interfaces, Department of Colloid Chemistry Am Mühlenberg 1 14476 Potsdam Germany bernhard.schmidt@mpikg.mpg.de; Soft Matter and Functional Materials, Helmholtz-Zentrum Berlin für Materialien und Energie 14109 Berlin Germany; State Key Laboratory of Fine Chemicals, Department of Polymer Science and Engineering, Dalian University of Technology Dalian 116024 China; Institute of Chemistry, University of Potsdam 14476 Potsdam Germany

## Abstract

Self-assembly of block copolymers is a significant area of polymer science. The self-assembly of completely water-soluble block copolymers is of particular interest, albeit a challenging task. In the present work the self-assembly of a linear-brush architecture block copolymer, namely poly(*N*-vinylpyrrolidone)-*b*-poly(oligoethylene glycol methacrylate) (PVP-*b*-POEGMA), in water is studied. Moreover, the assembled structures are crosslinked *via* α-CD host/guest complexation in a supramolecular way. The crosslinking shifts the equilibrium toward aggregate formation without switching off the dynamic equilibrium of double hydrophilic block copolymer (DHBC). As a consequence, the self-assembly efficiency is improved without extinguishing the unique DHBC self-assembly behavior. In addition, decrosslinking could be induced without a change in concentration by adding a competing complexation agent for α-CD. The self-assembly behavior was followed by DLS measurement, while the presence of the particles could be observed *via* cryo-TEM before and after crosslinking.

## Abstract 2

Self-assembly of the double hydrophilic block copolymer poly(*N*-vinylpyrrolidone)-*b*-poly(oligoethylene glycol methacrylate) and supramolecular crosslinking via *α*-cyclodextrin in water is presented.

## Graphical abstract

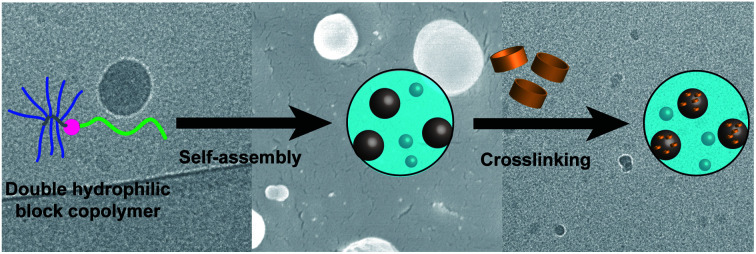

## Introduction

Block copolymer self-assembly has been in the focus of researchers in recent decades. In particular, amphiphilic block copolymers have gained significant attention for the purpose of self-assembly, *e.g.* in the formation of micelles,^1,2^ vesicles^3–5^ or more complex structures.^6,7^ Various applications are proposed for these kinds of structures, *e.g.* for drug-delivery,^8,9^ nano reactors,^10,11^ artificial cells^12^ or as templates for nanoparticles.^13^ Another option is to utilize a double hydrophilic block copolymer (DHBC) that features a stimuli responsive block. Such a stimuli responsive block is capable of switching from hydrophilic to hydrophobic upon application of heat,^14,15^ pH change^16,17^ or redox reactions^18,19^ to facilitate a self-assembly process. In this case the self-assembly is rather driven *via* hydrophobicity induced aggregation.

Recently, the self-assembly process could be extended towards completely hydrophilic block copolymers without the need of a triggered solubility change.^20^ The self-assembly can be understood from the formation of aqueous two phase systems from two hydrophilic homopolymers in water at elevated concentrations. The connection of both homo polymers – leading to a DHBC – diminishes macroscopic demixing but facilitates microscopic self-assembly. In such a way, various examples of DHBC self-assembled structures were described, *e.g.* vesicles,^21,22^ micelles^23–25^ or particles.^26–28^ In addition, mesophases of DHBCs in water were observed at high concentrations.^29,30^ For future applications the fact that DHBC-based self-assemblies are formed from completely water-soluble blocks might open up novel opportunities due to enhanced permeability in comparison to self-assemblies from amphiphilic block copolymers. In the case of amphiphilic block copolymers several strategies are utilized to tackle the permeability challenge, *e.g.* utilization of artificial protein channels^31^ or responsive swellable domains.^32,33^ In contrast, for DHBC self-assemblies such strategies might not be necessary to ensure permeability. Therefore, the resulting aggregates should be permeable towards water soluble compounds which make this class of material an excellent candidate for applications such as nanoreactors. Furthermore, there are more hydrophilic polymers proven to be FDA approved than hydrophobic polymers which allows the application of potential nanoreactors in the biomedical field as bioreactors.^34^

It could be shown that the self-assembly process depends on DHBC architecture, *e.g.* for a brush-linear DHBC enhanced self-assembly was observed compared to a linear–linear DHBC.^27^ Moreover, the efficiency of self-assembly depends significantly on the concentration of DHBC, and in general rather high concentrations are needed to drive self-assembly forward.^20,22^ The higher the concentration, the more favorable the state of aggregation is in comparison to the unimer state. Dilution of the DHBC solution results in an instant shift in the equilibrium causing the disassembly of the aggregates. Thus, stabilization of the aggregates towards changing concentration is a major challenge that has to be accomplished before approaching applications. In order to gain control over the equilibrium and stabilize the DHBC particles for further uses, it is inevitable to crosslink the DHBC particles as reported recently.^35,36^ In general, there are several crosslinking approaches: covalent, dynamic covalent and physical/supramolecular crosslinking. Covalent bonding is non-reversible, which enhances the long-term stability of crosslinks but excludes adaptivity of the system. On the other hand, dynamic covalent bonding allows further changes to the system or triggered cleavage without allowing instant changes. Finally, supramolecular crosslinking features reversibility as well as dynamic equilibration over the crosslinked structure. The focus in this study is to connect the dynamic nature of DHBC with the likewise dynamic nature of physical crosslinking approach.

A well-known way to crosslink polymers in aqueous solution is cyclodextrin (CD)-based host/guest chemistry,^37^*e.g.* for the formation of slide ring-gels,^38,39^ self-healing gels^40,41^ or stimuli-responsive gels.^42^ In particular α-cyclodextrin (α-CD) is widely used in the literature for the gelation of poly(ethylene glycol) (PEG) containing polymers.^43,44^ The crosslinking of α-CD and PEG is proceeding *via* inclusion complexation of α-CD and PEG followed by crystallization of the complexes and formation of crystalline domains that act as crosslinking points in aqueous solution.^45,46^ However, in this study no complete gelation of the solution is desired, but rather crosslinking in a defined space in the self-assembled DHBC-particles. In addition, CDs have been widely utilized in various applications, *e.g.* drug delivery,^47,48^ nano-structures,^43,49^ supramolecular polymers,^50,51^ amphiphiles^52,53^ or bioactive materials.^54,55^ Moreover, CD is a biocompatible and FDA approved compound which makes it highly interesting for research in the direction of biomedical applications. Recently, we could show the formation of supramolecular bulk hydrogels from α-CD and the DHBC poly(*N*-vinylpyrrolidone)-*b*-poly(oligoethylene glycol methacrylate) (PVP-*b*-POEGMA), which is related to the present work.^56^ The formed hydrogels featured remarkable thermoresponsive behavior, yet at increased concentration compared to the present work.

**Scheme 1:**
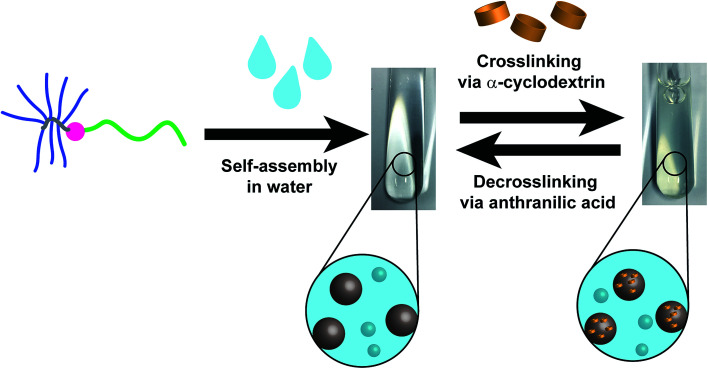
The self-assembly process of the linear-brush DHBC PVP-*b*-POEGMA in water followed by a crosslinking approach *via* α-cyclodextrin in order to physically crosslink the POEGMA-brush block. Finally, a decrosslinking process is induced by the addition of anthranilic acid (AA).

The focus of the present study lies on the effect of the crosslinking agent α-CD on the self-assembly behavior of the DHBC PVP-*b*-POEGMA (Scheme 1) with linear-brush architecture under diluted conditions ≤ 2.0 wt%, which is of significant difference to a linear-linear architecture both for self-assembly as well as synthesis. Thus, one of the goals of the study is to gain further pieces of information regarding linear-brush DHBC self-assembly. At first the linear-brush PVP-*b*-POEGMA DHBC is synthesized, then the self-assembly behavior is studied before and after the addition of different amounts of α-CD. The effect on self-assembly efficiency is studied *via* dynamic light scattering (DLS). Cryo-TEM and cryo-SEM are utilized to image the aggregates before and after crosslinking. Subsequently, the supramolecular nature of the crosslinking with α-CD is exploited by using anthranilic acid (AA) as a competitive guest compound for α-CD in order to induce decrosslinking.

## Experimental part

### Materials

Anthranilic acid (AA, 98+, Alfa Aesar), ammonium chloride (99%, Roth KG), ascorbic acid (98%, Alfa Aesar), 2-bromopropionyl bromide (97%, Sigma Aldrich), *t*-butyl hydroperoxide (70% solution in water, Acros Organics), chloromethyl polystyrene resin (2.4 mmol g^−1^, TCI), copper(i)bromide (CuBr, 99.99%, Sigma Aldrich), copper(ii)sulfate (CuSO_4_, 99%, Carl Roth), α-cyclodextrin (α-CD, >90%, Carl Roth), dichloromethane (DCM, analytical grade, Acros Organics), diethyl ether (ACS reagent, Sigma Aldrich), *N*,*N*-dimethylformamide (DMF, analytical grade, Sigma Aldrich), dimethylsulfoxide (DMSO, analytical grade, VWR Chemicals), 4,4′-dinonyl-2,2′-dipyridyl (dNBipy, 97%, Sigma Aldrich), ethyl acetate (EtOAc, analytical grade, Chem Solute), hexane (analytical grade, Fluka), hydrochloric acid (fuming, Carl Roth), magnesium sulfate (dried, Fisher Scientific), methanol (MeOH, analytical grade, Fisher Scientific), *N*,*N*,*N*′,*N*′′,*N*′′-pentamethyldiethylenetriamine (PMDETA, 98%, Sigma Aldrich), potassium-*O*-ethyl xanthate (98%, Alfa Aesar), propargyl alcohol (99%, Sigma Aldrich), pyridine (99% extra dry, Acros Organics), sodium azide (>99.5%, Fluka), sodium bicarbonate (>99%, Fluka), sodium sulfite (97%, Acros Organics) and triethylamine (99.5%, Sigma Aldrich) were used as received. *N*-Vinylpyrrolidone (VP, 99%, Sigma Aldrich) was dried over anhydrous magnesium sulfate and purified by distillation under reduced pressure. Oligo(ethylene glycol) methyl ether methacrylate (OEGMA, 900 g mol^−1^, Sigma Aldrich) was first dissolved in THF, then passed over a basic aluminum oxide column (Brockman I, Sigma Aldrich) and subsequently precipitated in cold hexane, filtered and dried under high vacuum for 24 h. Millipore water was obtained from an Integra UV plus pure water system by SG Water (Germany). Azido functionalized PS-resin (Fig. S1†), prop-2-yn-1-yl 2-((ethoxycarbonothioyl)thio) propanoate (alkyne-CTA), alkyne end functionalized PVP (Fig. S2†) azide end functionalized POEGMA (Fig. S3†) and PVP-*b*-POEGMA (Fig. S4–S8†) were prepared according to the literature.^21,27,56,57^ Spectra/Por dialysis tubes with MWCO of 10 000 were purchased from Spectrum Labs.

### Procedure for the preparation of solutions for DLS investigation

For the DLS measurement 50 mg of DHBC were dissolved in 2.5 g of Millipore water in order to receive a 2.0 wt% DHBC solution. Before conducting the DLS measurement the solution was passed through a 1.2 μm filter. The solutions containing 0.5 wt% and 0.1 wt% of DHBC were obtained by diluting the 2.0 wt% solution with water accordingly.

### Crosslinking of PVP-*b*-POEGMA *via* α-CD

In order to crosslink the PVP-*b*-POEGMA aggregates 500 mg of a 10.0 wt% DHBC solution were mixed with a certain amount of α-CD (25 mg, 50 mg, 75 mg, 100 mg corresponding to 5, 10, 15 and 20 wt% respectively). The mixture was stirred overnight and then diluted to 2.0 wt%. The 2.0 wt% solution was then utilized for several investigation methods, like cryo-SEM, cryo-TEM, and DLS. For the DLS measurement the 0.5 wt% and 0.1 wt% solutions could be obtained by diluting the 2 wt% solution accordingly.

### Decrosslinking of PVP-*b*-POEGMA *via* AA

In order to induce decrosslinking of the α-CD crosslinked PVP-*b*-POEGMA aggregates, first the DHBC was crosslinked with α-CD according to the procedure reported above. Subsequently, AA 15 mg, (1.05 : 1 molar ratio of AA : α−CD) were added to the PVP-*b*-POEGMA solution (2 wt%, 2.5 mL) and stirred overnight. Before DLS measurement the solution was passed through a 1.2 μm filter.

### Characterization methods

^1^H- and ^13^C-NMR spectra were recorded at ambient temperature at 400 MHz for ^1^H and 100 MHz for ^13^C with a Bruker Ascend400. Diffusion-ordered spectroscopy (DOSY) was performed at 600 MHz (Agilent Premium Shielded) with the dppste_cc pulse sequence. Dynamic light scattering (DLS) was performed using an ALV-7004 Multiple Tau Digital Correlator in combination with a CGS-3 Compact Goniometer and a HeNe laser (Polytec, 34 mW, *λ* = 633 nm at *θ* = 90° setup for DLS). Sample temperatures were adjusted to 25 °C. Toluene was used as immersion liquid. Apparent hydrodynamic radii (*R*_app_) were determined by LV-Correlator Software Version 3.0. Autocorrelation functions measured at a scattering angle of 90° were analyzed using cumulant and CONTIN methods. Cryogenic scanning electronic microscopy (cryo-SEM) was performed on a Jeol JSM 7500 F and the cryo-chamber from Gatan (Alto 2500). Size exclusion chromatography (SEC) for PVP, POEGMA and PVP-*b*-POEGMA were conducted in NMP (Fluka, GC grade) with 0.05 mol L^−1^ LiBr and BSME as internal standard at 70 °C using a column system with a PSS GRAM 100/1000 column (8 × 300 mm, 7 μm particle size), a PSS GRAM precolumn (8 × 50 mm), a Shodex RI-71 detector and a PMMA or PEO calibration with standards from PSS. Fourier transform infrared (FT-IR) spectra were acquired on a Nicolet iS 5 FT-IR spectrometer. Samples for cryogenic transmission electron microscopy (cryo-TEM) were prepared by applying a 4 μl droplet of sample suspension to lacey carbon copper grids (200 mesh, Science Services) and plunge frozen into liquid ethane using a Vitrobot Mark IV (FEI, Eindhoven, Netherlands) set at 4 °C and 95% humidity. The grids were mounted on a cryo transfer holder (Gatan 914, Gatan, Munich, Germany) and transferred into a JEOL JEM-2100 (JEOL GmbH, Eching, Germany) transmission electron microscope for imaging. The microscope was operated at an acceleration voltage of 200 kV and micrographs were recorded with a bottom-mounted 4 × 4k CMOS camera (TemCam-F416, TVIPS, Gauting, Germany) at a magnification of 50 000×, corresponding to a pixel size of 2.32 Å at the specimen level. Total electron dose for each micrograph was kept below 15 e^−^ Å^−2^.

## Results and discussion

### Synthesis and self-assembly of linear-brush PVP-*b*-POEGMA

As shown previously, PVP-*b*-POEGMA with linear-brush architecture can be synthesized *via* a two-step process. At first individual homopolymers were synthesized *via* RAFT polymerization and ATRP, respectively (Table S1†).^26,27^ Alkyne end functionalized PVP was synthesized *via* RAFT polymerization employing an alkyne functionalized chain transfer agent. In addition, azide end functionalized POEGMA was synthesized *via* ATRP with an azide functional initiator. Both polymers were characterized *via* SEC and NMR before further steps were performed (Fig. S2 and S3†). Unimodal SEC chromatograms were observed and *Đ* in the range of 1.08 to 1.43 (Table S1†). The molecular weights were varied to allow investigation of self-assembly according to molecular weights (38, 30, 24 and 14 kg mol^−1^ for PVP; 13 and 9 kg mol^−1^ for POEGMA). In order to obtain the DHBC, both blocks were coupled *via* copper catalyzed azide alkyne cycloaddition (CuAAc) according to the literature.^56^ To enable increased purity of block copolymer, an excess of the alkyne functional block was utilized and non-reacted amounts were removed *via* resin coupling followed by filtration. The linear-brush block copolymers were characterized *via* FTIR, SEC and NMR as well (Table S2, Fig. S4–S10†). In SEC, a clear shift towards lower elution volume was observed for example in comparison to the mixture of homopolymers (Fig. S4†). In addition, blends of block copolymer and homopolymers were investigated to ascertain the purity of the formed block copolymers. Minor differences in the elugrams of block copolymer sample and blends are observed even at homo polymer contents of 10 wt%, which indicates a purity of 90% or better for the block copolymers. Moreover, the presence of both blocks was observed in the NMR spectrum (Fig. S7†) and a similar diffusion coefficient was determined for both blocks in DOSY (diffusion-ordered spectroscopy) (Fig. S8†), which indicates block copolymer formation.

**Fig. 1:**
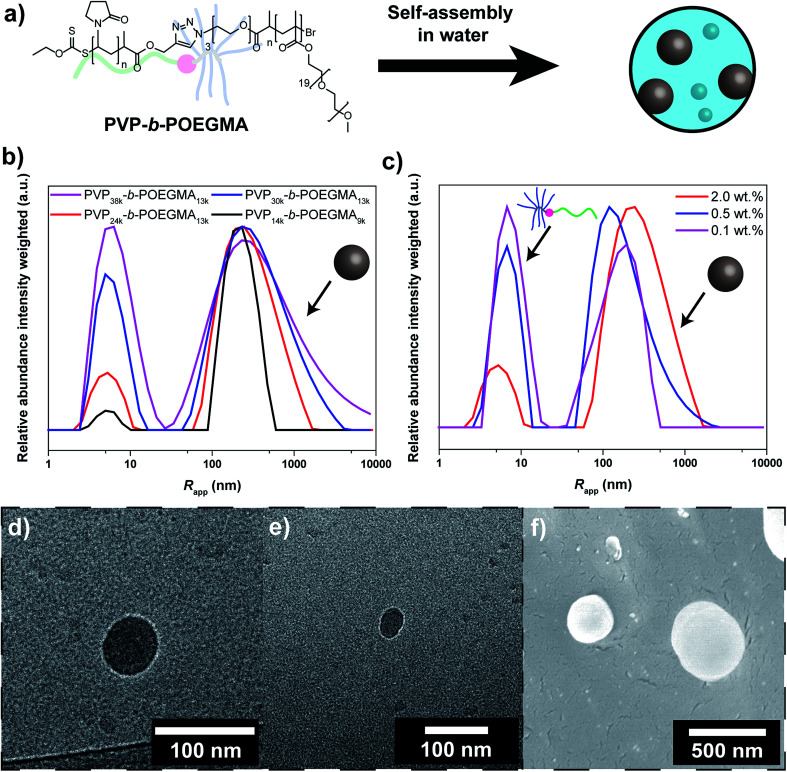
(a) Overview of the self-assembly process of the linear-brush DHBC, (b) intensity weighted particle size distribution of PVP_38k_-*b*-POEGMA_13k_ (magenta curve), PVP_30k_-*b*-POEGMA_13k_ (blue curve), PVP_24k_-*b*-POEGMA_13k_ (red curve) and PVP_14k_-*b*-POEGMA_9k_ (black curve) in Millipore water measured *via* DLS at 25 °C at a concentrations of 2.0 wt%, (c) intensity weighted particle size distribution of PVP_24k_-*b*-POEGMA_13k_ in Millipore water measured *via* DLS at 25 °C at different concentrations (red curve: 2.0 wt%, blue curve: 0.5 wt% and magenta curve: 0.1 wt%), (d and e) cryo-TEM images of PVP_24k_-*b*-POEGMA_13k_ at a concentration of 1.0 wt%, and (f) cryo-SEM images of PVP_24k_-*b*-POEGMA_13k_ at a concentration of 2.0 wt%.

Subsequently, self-assembly of the formed block copolymers was probed (Fig. 1a). Recently, it was shown that linear-brush block copolymers feature enhanced self-assembly compared to the linear-linear block copolymer analogues.^27^ At first DLS studies of the self-assembly/aggregation of the block copolymer were performed, suggesting particle sizes between 100 nm and 1 μm. The DLS results indicate a strong correlation between aggregation and *M*_n_ of the individual blocks (Fig. 1b). The efficiency increases with decreasing *M*_n_ of the PVP block from 38 to 24 kg mol^−1^ (at constant *M*_n_ of POEGMA of 13 kg mol^−1^). At shorter DP of both blocks, *i.e.* PVP_14k_-*b*-POEGMA_9k_, the efficiency is significantly enhanced. An explanation for the observed behavior might be the decreased steric demand of the blocks that facilitates aggregation. Moreover, PVP-*b*-POEGMA shows a very strong correlation between polymer concentration and self-assembly efficiency as observed in the case of PVP_24k_-*b*-POEGMA_13k_ (Fig. 1c). A significant decrease of self-assembled structures is observed upon dilution of the DHBC solution as expected from literature.^35^ It should be noted that intensity averaged particle distributions are plotted, where larger structures are overexpressed.

To further elucidate the self-assembly process and support the observations from DLS, cryo-TEM studies were performed (Fig. 1d/e). Circular structures with sizes in the range of 30–50 nm can be observed under cryo-TEM, which indicates the presence of spherical particles in solution. In addition to spherical particles, particles with less defined structures with sizes in the range of 20–100 nm can be observed as well that are most likely agglomerates (Fig. S11†). Moreover, cryo-SEM was performed that shows spherical particles as well. Particle sizes between 50 and 500 nm are observed, which matches the results from DLS (Fig. 1f).

### Crosslinking of linear-brush PVP-*b*-POEGMA *via* α-cyclodextrin (α-CD)

In the next step, crosslinking of PVP_24k_-*b*-POEGMA_13k_ with α-CD was investigated. Therefore, a 10.0 wt% solution of PVP_24k_-*b*-POEGMA_13k_ was prepared and various amounts of α-CD were added. The final solution was stirred over-night and diluted to reach a final DHBC concentration of 2.0 wt% while the α-CD concentration was varied between 4.0 wt%, 3.0 wt%, 2.0 wt%, and 1.0 wt% respectively. In the optimization of the crosslinking process caution was taken to ensure particles perform intra particle crosslinking only. The presented procedure features crosslinking at higher DHBC concentrations, which is a common strategy for DHBC systems as self-assembly efficiency strongly depends on concentration. In such a way, self-assemblies can be crosslinked and preserved in diluted conditions.

**Fig. 2:**
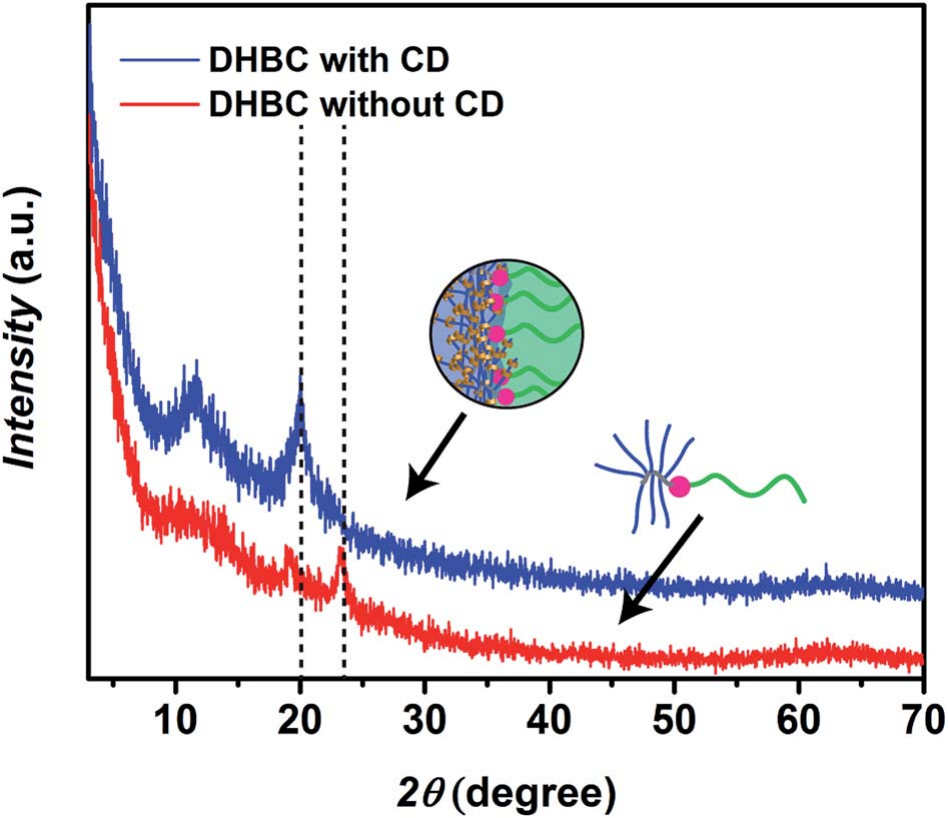
X-ray diffraction (XRD) of freeze-dried samples of the linear-brush DHBC PVP_24k_-*b*-POEGMA_13k_ before crosslinking at a concentration of 2.0 wt% (red curve) and after crosslinking with α-CD at a DHBC and α-CD concentration of 2.0 wt% (blue curve).

It is well known that α-CD and PEG form crystalline inclusion complexes which can be illustrated as necklace-like supramolecular structures.^58^ Therefore, the α-CD crosslinked DHBC was freeze-dried and the crystal formation was studied *via* X-ray diffraction (XRD). As a result a relevant peak could be identified at 20.1° (0.44 nm), which can be assigned to the [210] reflex of hexagonal lattices with *a* = 13.6 Å and resembles the α-CD core in a channel-like organization.^59^ The peak was absent prior crosslinking indicating the formation of α-CD/POEGMA crystalline structures. The reflexes at 24° in the native DHBC are due to the semi-crystalline nature of the POEGMA block that disappear in the crosslinked product as POEGMA crystallization is hindered after α-CD complexation (Fig. 2).

**Fig. 3:**
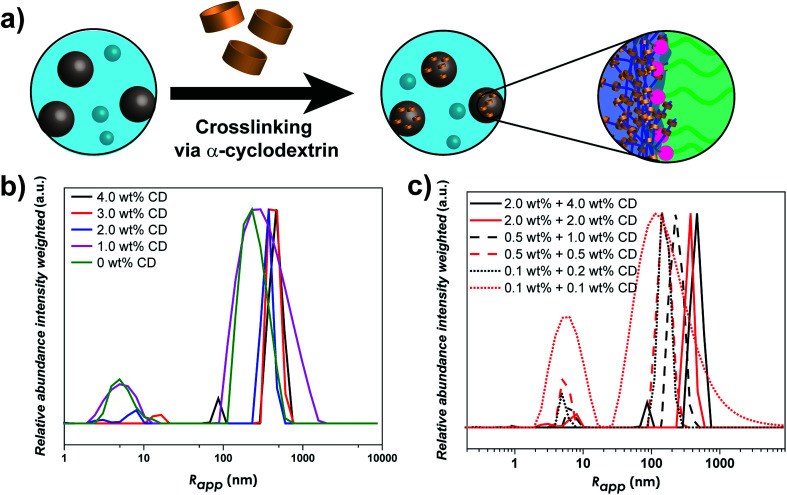
(a) Schematic overview of the crosslinking process of the linear-brush DHBC, (b) intensity weighted particle size distribution of 2.0 wt% PVP_24k_-*b*-POEGMA_13k_ in Millipore water and the addition of α-CD (4.0 wt%, 3.0 wt%, 2.0 wt%, 1.0 wt% and 0 wt%) measured *via* DLS at 25 °C and (c) intensity weighted particle size distribution of PVP_24k_-*b*-POEGMA_13k_ in Millipore water with six α-CD (4.0 wt% to 0.1 wt%) and three DHBC concentrations (2.0 wt%; 0.5 wt%; 0.1 wt%) measured *via* DLS at 25 °C.

As linear-brush PVP-*b*-POEGMA shows a very strong correlation between polymer concentration and self-assembly efficiency, changes in the self-assembly behavior upon α-CD addition were investigated. In order to study the effect of α-CD on the self-assembly behavior of PVP_24k_-*b*-POEGMA_13k_ five different samples were prepared to correlate α-CD content and self-assembly behavior. Each sample contains 2.0 wt% aqueous DHBC solution with a different amount of α-CD (4.0 wt% α-CD, 3.0 wt% α-CD, 2.0 wt% α-CD, 1.0 wt% α-CD and 0 wt% α-CD) and then studied *via* DLS measurements. The dynamic nature of the self-assembly behavior of pure PVP-*b*-POEGMA could be already illustrated *via* DLS measurements, as polymer concentration affects strongly the self-assembly efficiency. The higher the DHBC concentration, the more the ratio shifted from unimers towards aggregate formation. Adding α-CD which itself builds a dynamic non-covalent interaction results in a highly tunable, predictable and unique property for DHBC (Fig. 3b).

The main effect of α-CD addition on the self-assembly behavior follows the same trend in all concentration ranges, the stepwise increase of α-CD results in a stepwise increase in the self-assembly efficiency. Considering the self-assembly behavior of DHBC at 2.0 wt%, the addition of α-CD shows a clear improvement in the ratio of unimers fraction to aggregate formation (Fig. 3b). While the addition of 1.0 wt% α-CD has only a slight impact on the self-assembly process, as the normalized intensity of the unimer fraction decreases from 0.18 to 0.16 (Table S3†), the further addition of 2.0 wt% α-CD results in a substantial decrease to 0.06. This significant change in the self-assembly behavior can also be seen by the increase of the particle size of the small sized fraction. While pure DHBC shows a signal at 5.0 nm that can be interpreted as an unimer fraction, the signal shifts to 8.0 after the addition of 2.0 wt% α-CD indicating that the solution contains already polymer associates instead of free polymers. Further decrease of the unimer fraction can be observed when the amount of α-CD is increased. After addition of 3.0 wt% α-CD unimer species are absent and a transition to a system of small probably micellar aggregates (size around 17 nm) and large aggregates around 374 nm are indicated *via* DLS. Finally, after the addition of 4.0 wt% α-CD the first aggregate peak shifts from 17 to 86 nm, which represents clearly the formation of another aggregate species probably an aggregate of micelles. Thus, at an α-CD content of 3 wt% and 4 wt% the unimers fraction is diminished in the intensity weighted DLS measurement indicating a complete crosslinking. Hence, no unimers are found for 3 and 4 wt% α-CD but small aggregates, which probably represent micelles and aggregates of micelles. Moreover, the particle fraction that was present from the beginning also shows a stepwise increase in size with the increased addition of α-CD, starting from *R*_app_ = 230 nm without any α-CD to a *R*_app_ = 463 nm when 4.0 wt% α-CD is added (Table S3†).

In the next step, the self-assembly behavior after α-CD addition was probed upon dilution to allow the investigation of the dynamic equilibrium between unimer fraction and aggregates formation. First the solutions were diluted to 0.5 wt% and further to 0.1 wt% DHBC content. The self-assembly behavior in the other two concentration regions (0.5 wt% and 0.1 wt%) shows some similarities but also differences. While the amount of free polymer does decrease after each addition of α-CD stepwise (Fig. S12†) indicating that α-CD is inducing a self-assembly of the DHBC, there is still a noticeable amount of unimer fraction even after a starting concentration of 4.0 wt% α-CD (final concentration 1.0 wt% at 0.5 wt% DHBC and 0.2 wt% at 0.1 wt% DHBC). Conclusively, the α-CD induced self-assembly process at 2.0 wt% is partially reversible when the sample is diluted, which strongly differs from other previous reported DHBC crosslinking systems where the crosslinking agent could stabilize the particles against dilution.^21,36^ In contrast, the present supramolecular crosslinking strategy introduces an additional equilibrium to enhance the self-assembly process but sustaining the dynamics of the DHBC system.

The stepwise decrease in the unimers fraction can also be observed when the solution is further diluted to 0.1 wt% DHBC (Fig. S8†), as the intensity decreases steadily from 0.82 when no α-CD was present to 0.16 after the addition at 0.2 wt% of α-CD. It is thus noteworthy that the highly diluted DHBC solution (0.1 wt%) with 0.2 wt% of α-CD (Table S3†) shows a better self-assembly efficiency than the initial pure DHBC solution at 2.0 wt% (Table S3†). Therefore, the addition of 4.0 wt% α-CD can overcompensate the concentration-depended decrease in self-assembly efficiency.

Fig. 3c compares different concentration ranges with similar amounts of α-CD. Here, an interesting trend can be observed. α-CD does not only affect the self-assembly efficiency by decreasing the amount of unimer fraction but it has a significant impact on the particle size of the aggregates. When keeping the amount of α-CD constant, a reduction in polymer concentration leads to a decrease in particle size in all cases. Using 4.0 wt% α-CD as a starting point a decrease from *R*_app_ = 463 nm (2.0 wt%) to *R*_app_ = 226 nm (0.5 wt%) is observed and, finally *R*_app_ = 140 nm at a polymer concentration of 0.1 wt%. Moreover, within a certain polymer concentration the amount of α-CD has also a significant effect on the particle size. The more α-CD was used the larger particles could be obtained. A higher concentration of α-CD leads to larger particles, as shown for the case of 2.0 wt% and 4.0 wt% of α-CD in Fig. 3c.

**Fig. 4:**
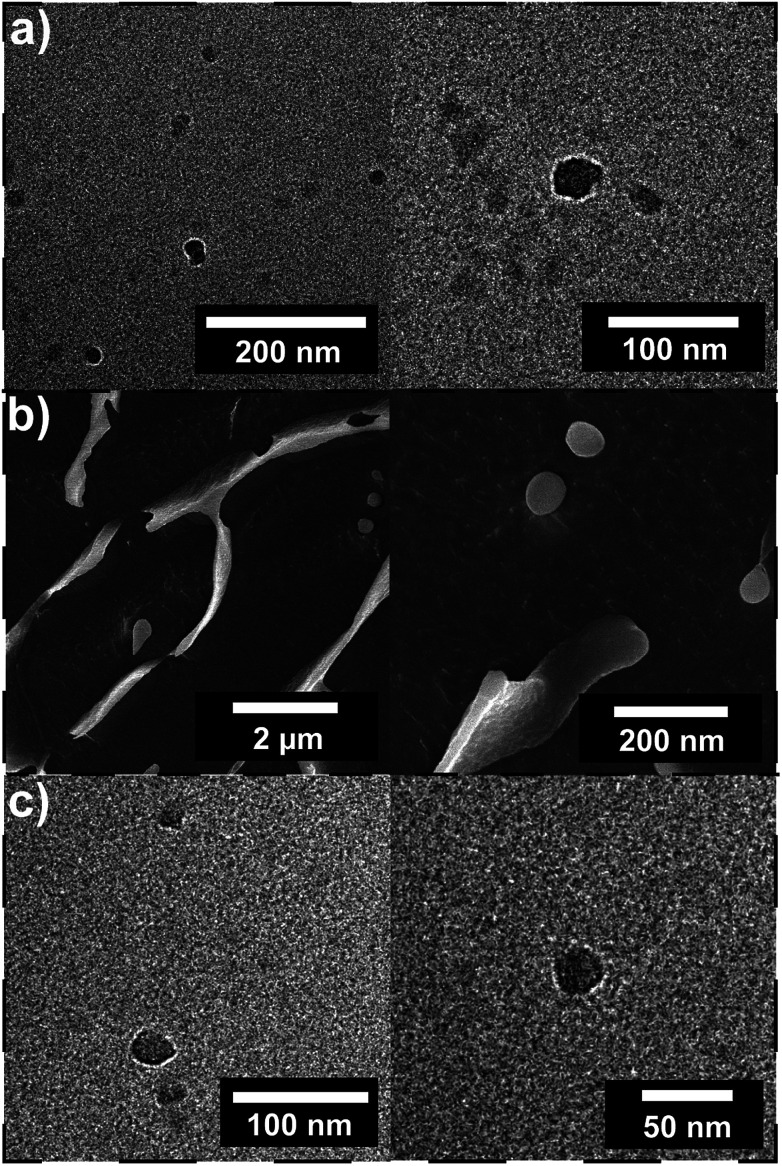
(a) Cryo-TEM images of PVP_24k_-*b*-POEGMA_13k_ at a concentration of 2.0 wt% crosslinked with 2.0 wt% α-CD, (b) cryo-SEM images of PVP_24k_-*b*-POEGMA_13k_ at a concentration of 2.0 wt% crosslinked with 2.0 wt% α-CD and (c) cryo-TEM images of PVP_24k_-*b*-POEGMA_13k_ at a concentration of 2.0 wt% crosslinked with 4.0 wt% α-CD.

In order to study the α-CD stabilized aggregates in more detail, cryo-TEM and cryo-SEM were performed. The presence of particles could be proven after the addition of 2.0 wt% and 4.0 wt% of α-CD. Also after the addition of α-CD spherical particles in the size range of 30–50 nm could be observed (Fig. 4a/c). In addition, polymer particles in the range of 3–4 nm were observed that can be attributed to remaining unimers. However, the particles observed after the addition of α-CD show a smaller particle size than indicated by the DLS measurement. This may be due to the reason that the DHBC does not build a dense shell in contrast to amphiphilic block copolymers due to the water solubility of both blocks. Furthermore, cryo-SEM showed spherical particles in the range of 50 to 500 nm (Fig. 4b), which is in line with DLS measurements.

### Decrosslinking of the linear-brush DHBC aggregates

**Fig. 5:**
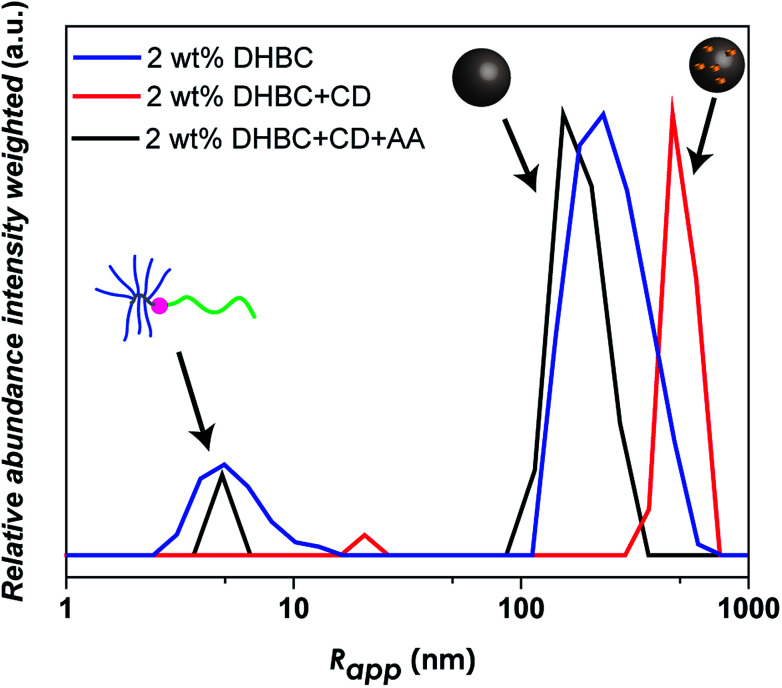
Comparison of intensity weighted particle size distribution measured *via* DLS at 25 °C at different concentrations of the linear-brush PVP_24k_-*b*-POEGMA_13k_ before and after the crosslinking procedure with 4.0 wt% α-CD and the subsequent decrosslinking process *via* anthranilic acid (AA) at 2.0 wt% DHBC.

The reversal of crosslinking is a very interesting process, as long as it is controlled and induced in purpose in order to breakdown the aggregates after the fulfillment of the desired function. Especially in the biomedical field, it is inevitable to introduce a certain mechanism of decrosslinking, otherwise the aggregates could accumulate in the body. For this reason, the α-CD crosslinked linear-brush PVP_24k_-*b*-POEGMA_13k_ particles were treated with anthranilic acid (AA) as a competitive guest compound for α-CD. AA shows a high affinity towards α-CD,^60^ while it is a natural product and sometimes referred to as vitamin L_1_. However, after adding AA to a 2.0 wt% DHBC solution containing 4.0 wt% of α-CD, decrosslinking can be observed (Fig. 5). Comparing the disassembled sample with the crosslinked DHBC and the initial sample before crosslinking a significant change could be observed. The addition of AA induces decrosslinking as indicated by a significant fraction of unimers after the addition of AA, as AA is successfully competing with POEGMA for the complexation with α-CD. However, AA is not able to complex all of α-CD, although AA was added with a ratio of 2 : 1 (AA : CD), as the dilution to 0.5 wt% (Fig. S13†) and 0.1 wt% (Fig. S13†) shows a substantial difference in the amount of unimer fraction compared to the DHBC sample before crosslinking.

## Conclusions

In the present work, we could show the successful crosslinking of linear-brush PVP-*b*-POEGMA *via* α-CD. Moreover, α-CD presents an interesting crosslinking behavior that shifts the equilibrium toward aggregate formation without switching off the dynamic equilibrium of linear-brush DHBC. As a consequence, the self-assembly efficiency is improved without extinguishing the unique DHBC self-assembly behavior, as dilution still causes a small but noteworthy shift in the equilibrium towards decrosslinking. Moreover, decrosslinking could also be induced without a change in concentration by adding AA as a competing complexation agent for α-CD. The self-assembly behavior was followed by DLS measurement, while the presence of the particles could be observed *via* cryo-TEM before and after crosslinking. These aggregate structures might be of interest for various applications in the future including enzymatic reaction environments and encapsulation.

## Conflicts of interest

There are no conflicts to declare.

## Supplementary Material

RA-009-C8RA10672J-s001
