# Supplementary material for: Poly(ethylene glycol) brush-b-poly(N-vinylpyrrolidone)-based double hydrophilic block copolymer particles crosslinked via crystalline α-cyclodextrin domains
Source: RSC Adv. 2019 Feb 8;9(9):4993–5001. doi: 10.1039/c8ra10672j (PMC9060675; doi:10.1039/c8ra10672j)
Supplement: RA-009-C8RA10672J-s001 [file RA-009-C8RA10672J-s001.pdf]

Electronic Supplementary Material (ESI) for RSC Advances.
This journal is © The Royal Society of Chemistry 2019

Supporting Information:

Poly(ethylene glycol) brush-*b*-poly(*N*-vinylpyrrolidone)-based double hydrophilic block copolymer particles crosslinked via crystalline α-cyclodextrin domains

Noah Al Nakeeb,[†] Zdravko Kochovski,[ǂ] Tingting Li,[†,‖,|] Youjia Zhang,[†] Yan Lu,[ǂ,‡] Bernhard V. K. J. Schmidt[†,*]

[†] Max-Planck Institute of Colloids and Interfaces; Department of Colloid Chemistry, Am Mühlenberg 1, 14476 Potsdam, Germany

ǂ Soft Matter and Functional Materials, Helmholtz-Zentrum Berlin für Materialien und Energie, 14109 Berlin, Germany

‖ State Key Laboratory of Fine Chemicals, Department of Polymer Science and Engineering, Dalian University of Technology, Dalian, 116024, China

‡ Institute of Chemistry, University of Potsdam, 14467 Potsdam, Germany

Email: bernhard.schmidt@mpikg.mpg.de

| Author present address: Academy of Advanced Battery Materials, Division 2, BYD Company Limited, Baoping Road No.1, Longgang, Shenzhen 518116, China

Additional Synthetic Procedures and Analytical Data

Prop-2-yn-1-yl 2-bromopropanoate[1]

In a dry, argon purged 250 mL Schlenk tube, propargyl alcohol (3.94 g, 4.1 mL, 70.2 mmol, 1.0 eq.) and triethylamine (9.95 g, 13.6 mL, 98.3 mmol, 1.4 eq.) were dissolved in dry THF (150 mL). The reaction mixture was cooled to 0 °C and 2-bromopropionyl bromide (18.19 g, 8.83 mL, 84.24 mmol, 1.2 eq.) was added slowly dropwise to the reaction mixture. The reaction mixture was allowed to warm to ambient temperature and stirred for 5 hours at ambient temperature. The formed salt was filtered off and the organic phase was subsequently washed with 2 M HCl solution (3 × 10 mL), deionized water (3 × 30 mL), saturated $NaHCO_3$ solution (3 × 10 mL), and deionized water (3 × 30 mL). The organic phase was dried over anhydrous $MgSO_4$ and the solvent was removed under reduced pressure. The crude product was purified via vacuum distillation (98 °C, 37 mbar) to afford prop-2-yn-1-yl 2-bromopropanoate (8.0 g, 42.11 mmol, 60% yield) as slightly yellow liquid.

^{1}H NMR (400 MHz, $CDCl_3$ δ:) 4.76 (dd, ^1J = 3.8 Hz, ^4J = 2.5 Hz 2H, CH_2), 4.40 (q, ^3J = 6.9 Hz, 1H, *CH*), 2.52 (t, ^4J = 2.5 Hz, 1H, *alkyne-H*), 1.84 (d, ^3J = 6.9 Hz, 3H, CH_3) ppm.

Prop-2-yn-1-yl 2-((ethoxycarbonothioyl)thio) propanoate[1]

In a dry, argon purged 250 mL Schlenk flask, prop-2-yn-1-yl 2-bromopropanoate (3.0 g, 15.79 mmol, 1.0 eq.) was dissolved in dry THF (200 mL). Potassium *O*-ethyl xanthate (25.78 g, 157.93 mmol, 10.0 eq.) was added portion wise to the solution under argon flow. The reaction mixture was stirred over night at ambient temperature. The formed salt and the excess of

potassium *O*-ethyl xanthate was filtered off and the organic phase was washed with deionized water (4 × 75 mL) and dried over anhydrous $MgSO_4$. The solvent was evaporated to afford prop-2-yn-1-yl 2-((ethoxycarbonothioyl)thio) propanoate (3.145 g, 13.55 mmol, 86% recovery) as yellow oil.

^{1}H NMR (400 MHz, $CDCl_3$ δ:) 4.74 (d, ^4J = 2.5 Hz 2H, *CH_2*), 4.61 (q, ^3J = 7.1 Hz, 2H, *CH_2O*), 4.41 (q, ^3J = 7.4 Hz, 1H, *CH*), 2.52 (t, ^4J = 2.5 Hz, 1H, *alkyne-H*), 1.58 (d, ^3J = 7.4 Hz, 3H, *CH_3*) 1.42 (t, ^3J = 7.1 Hz, 3H, *CH_3*) ppm.

Synthesis of azidomethyl polystyrene resin[2]

In a dry, argon purged 100 mL round bottom Schlenk tube chloromethyl polystyrene resin (10.0 g, 24.0 mmol, 1.0 eq.) was dissolved in dry DMSO (50 mL). Sodium iodide (10.8 g, 72.0 mmol, 3.0 eq.) and sodium azide (15.6 g, 240.0 mmol, 10.0 eq.) were added and the reaction mixture was moderately stirred for 48 hours at 80 °C. The afforded resin was filtered over a glass frit (pore size 3) and alternately washed with DCM (6 × 30 mL) and MeOH (6 × 30 mL). The purified resin was finally washed with diethyl ether (30 mL) and dried under vacuum to afford azidomethyl polystyrene resin (9.25 g, 22.2 mmol, 93% recovery) as a white solid. FT-IR ($\tilde{\nu}$): 3120, 2855, 2089 (N_3), 1509, 1450, 750, 697 cm^{-1}.

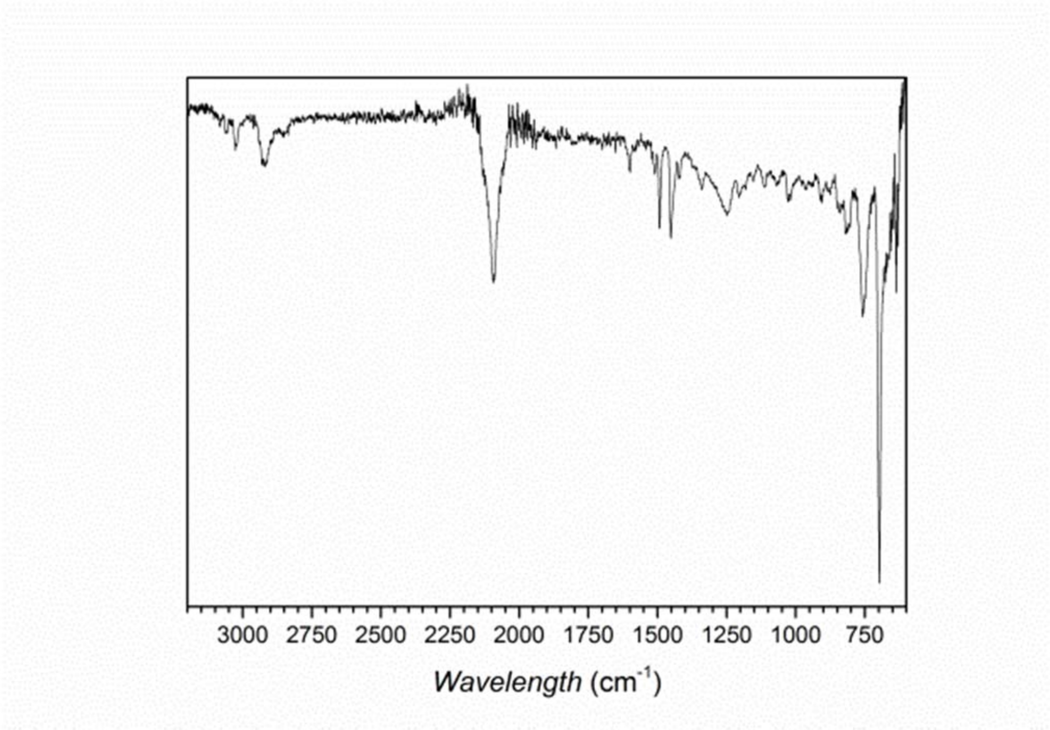

Figure S1. FT-IR spectrum of azidomethyl polystyrene resin recorded at 25 °C.

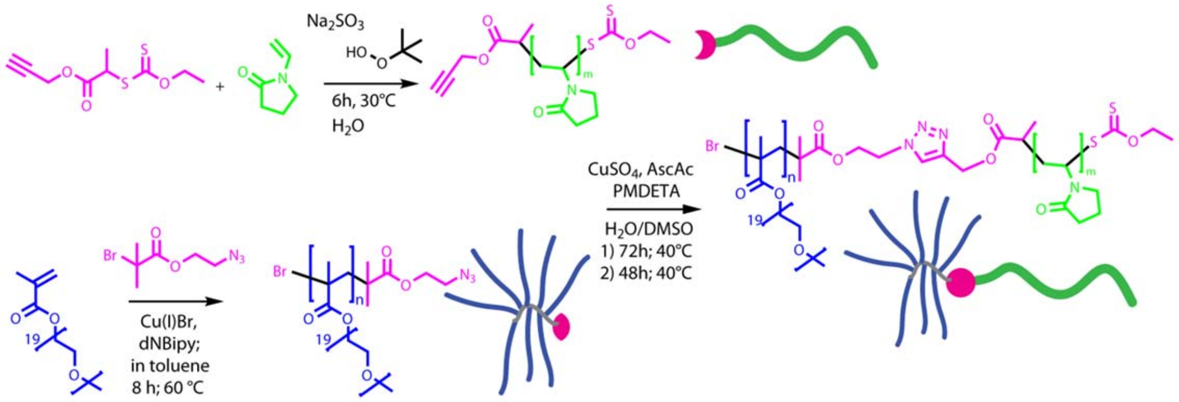

Scheme S1. (a) RAFT polymerization of *N*-vinylpyrrolidone via alkyne functionalized chain transfer agent. (b) ATRP of OEGMA via AzTEGBr to achieve the corresponding P(OEGMA)-

brush polymer block. (c) Copper(I) catalyzed cycloaddition of the P(OEGMA)-brush polymer and the alkyne terminated PVP to afford the PVP-*b*-P(OEGMA) linear-brush block copolymer.

Synthesis of PVP$_{14k/24k/30k/38k}$

In a dry, Ar purged 25 mL Schlenk tube, prop-2-yn-1-yl 2-((ethoxycarbonothioyl)thio)propanoate (0.0175 g, 0.075 mmol, 1.0 eq.) was dissolved in deionized water (3.36 mL). VP (4.17 g, 37.5 mmol, 500 eq.) and *t*-BuOOH solution (2.9 mg of 70 wt.% solution, 0.0225 mmol, 0.3 eq.) were added to the solution. Then the mixture was degassed via three freeze, pump and thaw cycles followed by a last freeze and purging with Ar. While purging with Argon, sodium sulfite (3.8 mg, 0.03 mmol, 0.2 eq.) was added. The reaction mixture was then immersed in an oil bath at 25 °C. After 2 h, the polymerization was quenched with liquid N_2 and exposed to air. Water was removed via reduced pressure and the crude polymer was dissolved in a small amount of MeOH. The mixture was precipitated twice into cold diethyl ether to afford alkyne terminated PVP (PVP-alkyne) as a white powder. (Yield 1.90 g, 50% recovery, $M_{n,SEC}$ = 24 100 g mol^{-1} (PMMA equivalents in NMP), $Đ$ = 1.29).

In order to synthesize PVP with different M_n the reaction time was varied while keeping all the ratio constant. After 1h of reaction time PVP$_{14k}$ could be achieved, while expanding the reaction time to 2 h resulted in PVP$_{24k,}$ 2.5h in PVP$_{30k}$ and PVP$_{38k}$ was achieved after 3 hours reaction time.

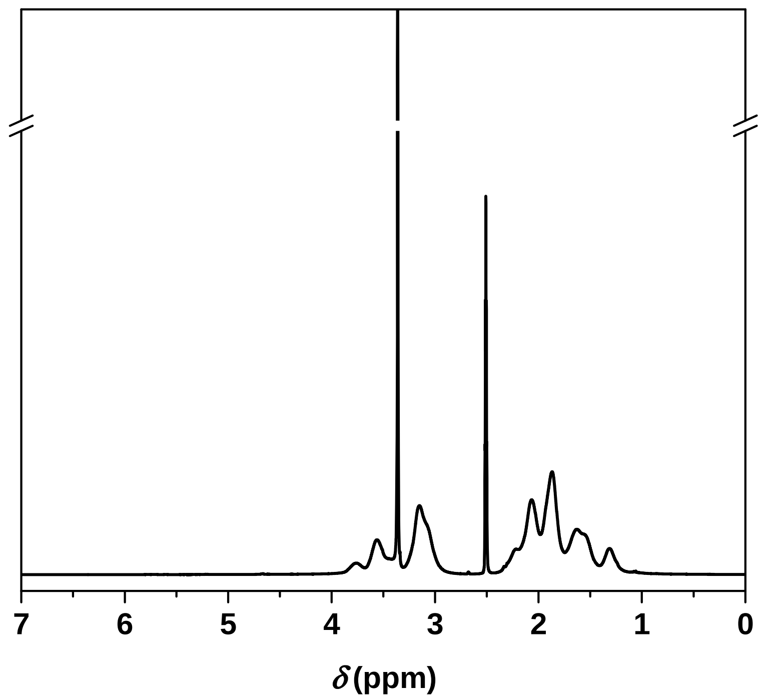

Figure S2. ¹H NMR spectrum of PVP_{24k} recorded at 400°MHz in DMSO-d_6.

Table S1. $M_{n,SEC}$ and $Đ$ of each PVP measured in NMP-SEC using PMMA calibration.

linear-polymer	$M_{n,SEC}$ [g mol^{-1}]	$Đ$
PVP_{14k}	13800	1.34
PVP_{24k}	24100	1.29
PVP_{30k}	30700	1.43
PVP_{38k}	38000	1.38

Synthesis of POEGMA$_{13k/9k}$

A stirring bar, 2-azidoethyl 2-bromoisobutyrate (0.047 g, 0.2 mmol, 1 eq), dNbpy (0.160 g, 0.4 mmol, 2 eq), destabilized OEGMA (4.75 g, 5 mmol, 20 eq) and toluene (11 mL, 9.5 g) were placed in a Schlenk tube. The tube was first sealed with a septum then the mixture was degassed via three freeze, pump and thaw cycles followed by a last freeze and purging with Ar. While purging with Argon, CuBr (0.028 mg, 0.2 mmol, 1 eq) was added to the frozen mixture and the Schlenk tube was purged with Ar until the frozen solution thawed and the solution color changed to brown indicating the formation of the copper-complex. Subsequently, the reaction mixture was placed in an oil bath and stirred at 60 °C for 6 hours. The reaction was cooled to ambient temperature and the reaction was stopped by removing the septum allowing oxygen to enter the mixture. The residue was dissolved in THF and passed through a short column filled with neutral aluminum oxide. The resulting solution was concentrated and the polymers precipitated in cold hexane. Subsequently the solution was filtered and was dialyzed against water (cut-off: 10 kDa) for three days. Subsequently the solution was lyophilized to afford POEGMA as a colorless solid (Yield 1.90 g, 41% recovery, $M_{n,SEC}$ = 13 200 g mol^{-1} (PEO equivalents in NMP), $Đ$ = 1.08).

In order to achieve the shorter POEGMA$_{9k}$ the reaction time was reduced to 4 hours. (Yield 1.40 g, 29% recovery, $M_{n,SEC}$ = 9 000 g mol^{-1} (PEO equivalents in NMP), $Đ$ = 1.12).

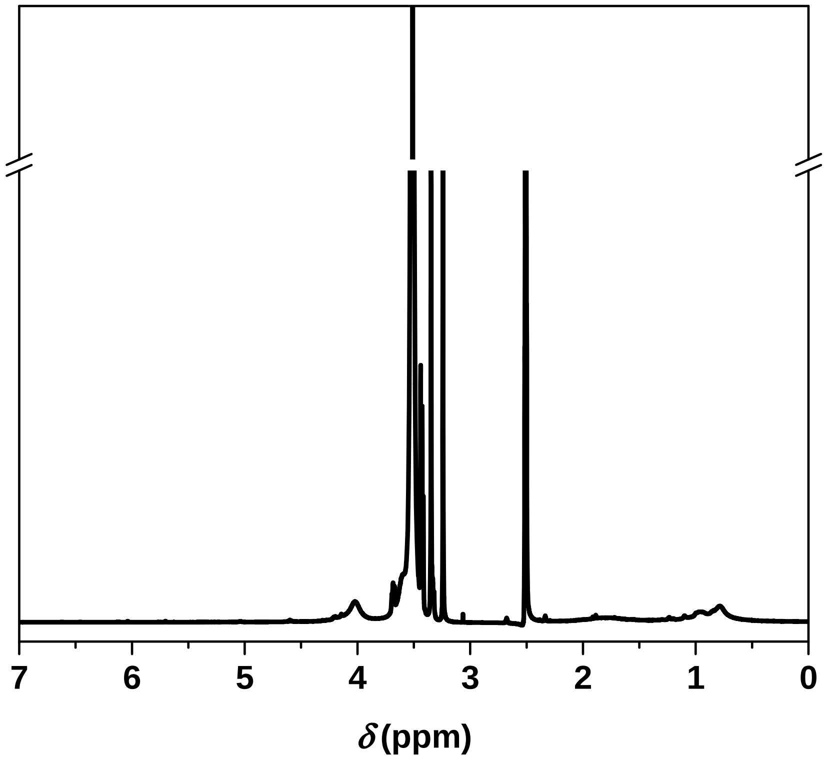

Figure S3. ^{1}H NMR spectrum of $POEGMA_{13k}$ recorded at 400°MHz in DMSO-d_6.

Synthesis of PVP_{24k}-b-$POEGMA_{13k}$

In a dry, argon purged 25 mL round bottom Schlenk flask, alkyne end functionalized PVP (1.63 g, 0.048 mmol, 1.2 eq.) was dissolved in deionized water (10.0 mL). $CuSO_4$ (4.2 mg, 26.0 µmol, 0.65 eq.) and DMSO (10.0 mL) were added to the solution. Azide end functionalized POEGMA (0.84 g, 0.04 mmol, 1.0 eq.) and PMDETA (0.01 g, 0.06 mmol, 1.5 eq.) were dissolved in DMSO (2.0 mL) and added to the reaction mixture. Finally, ascorbic acid (14.0 mg, 0.08 mmol, 2.0 eq.) was added twice, once directly at the beginning of the reaction then after 24 hours. The reaction mixture was stirred at ambient temperature for 48 hours. Azido

functionalized PS-Resin (21.3 mg, 0.048 mmol) and ascorbic acid (14.0 mg, 0.08 mmol, 2.0 eq.) were added and the reaction mixture was stirred for additional 48 h. The resin was filtered off and the solution was dialyzed against deionized water for three days followed by lyophilization to afford PVP-*b*-POEGMA (1.75 g, 69% recovery M_n = 51 000 g·mol^{-1}, PMMA standard in NMP, $Đ$ = 1.29) as a white powder.

The other blocks were synthesized in the same way keeping the equivalents constant except for the monomer amount. In Table S2 the SEC results for each DHBC are summarized.

Table S2. $M_{n,SEC}$ and $Đ$ of each DHBC measured in NMP-SEC using PMMA calibration.

**linear-polymer**	$M_{n,SEC}$ **[g mol^{-1}]**	$Đ$
PVP_{14k}-*b*-$POEGMA_{9k}$	38800	1.30
PVP_{24k}-*b*-$POEGMA_{13k}$	51100	1.29
PVP_{30k}-*b*-$POEGMA_{13k}$	50900	1.42
PVP_{38k}-*b*-$POEGMA_{13k}$	59100	1.54

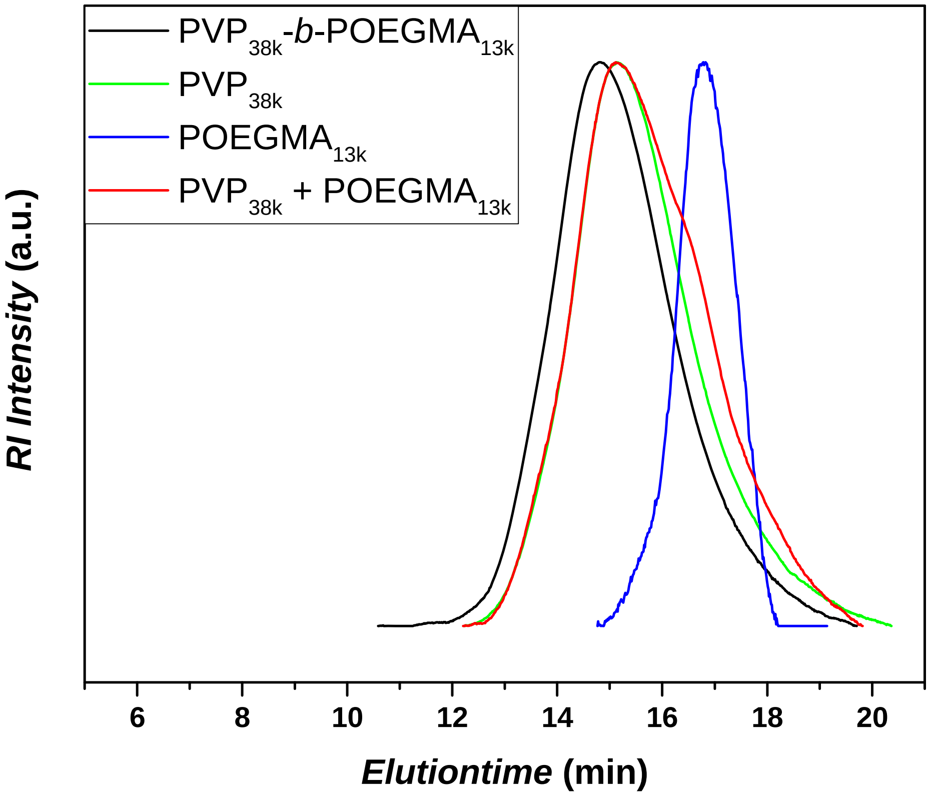

Figure S4. SEC elugrams of PVP_{38k}-*b*-$POEGMA_{13k}$ block copolymer and constituents measured in NMP.

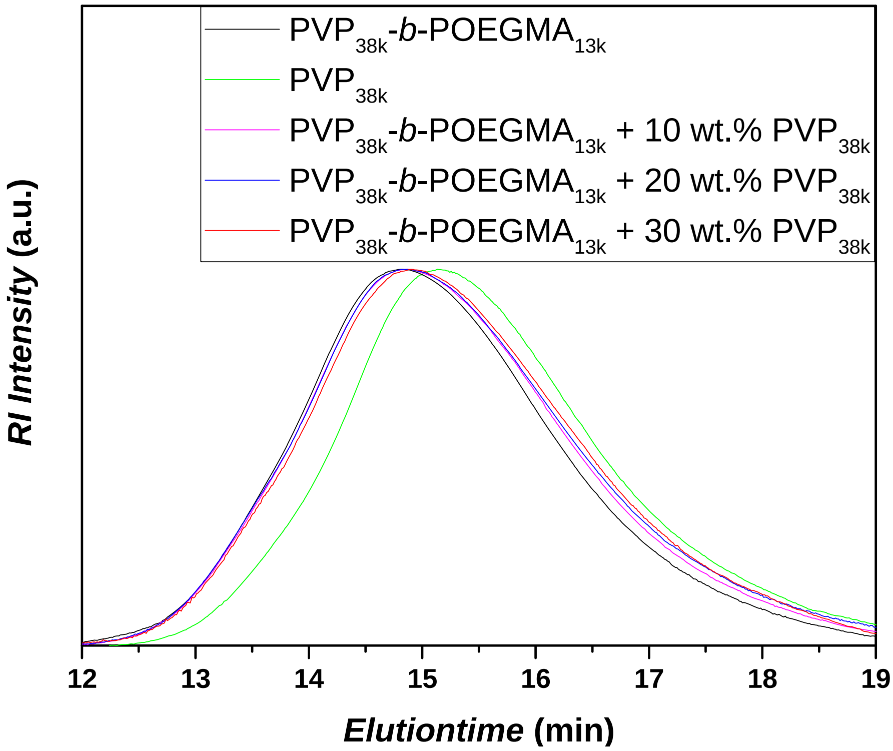

Figure S5. SEC elugrams of PVP_{38k}-*b*-$POEGMA_{13k}$ block copolymer and blends with PVP_{38k} measured in NMP.

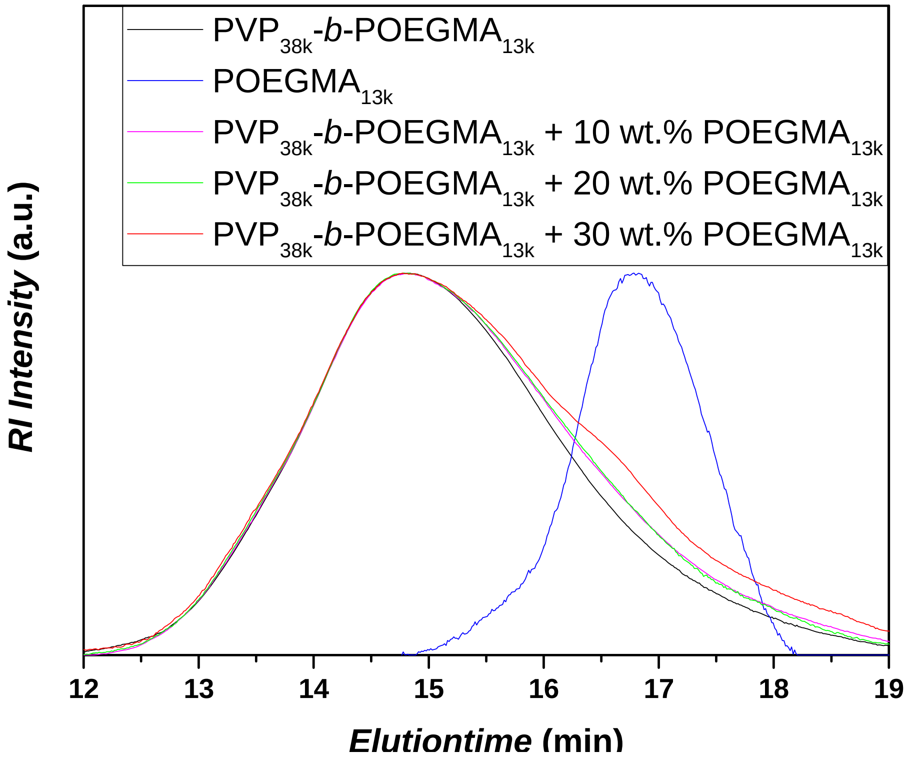

Figure S6. SEC elugrams of PVP_{38k}-*b*-$POEGMA_{13k}$ block copolymer and blends with $POEGMA_{13k}$ measured in NMP.

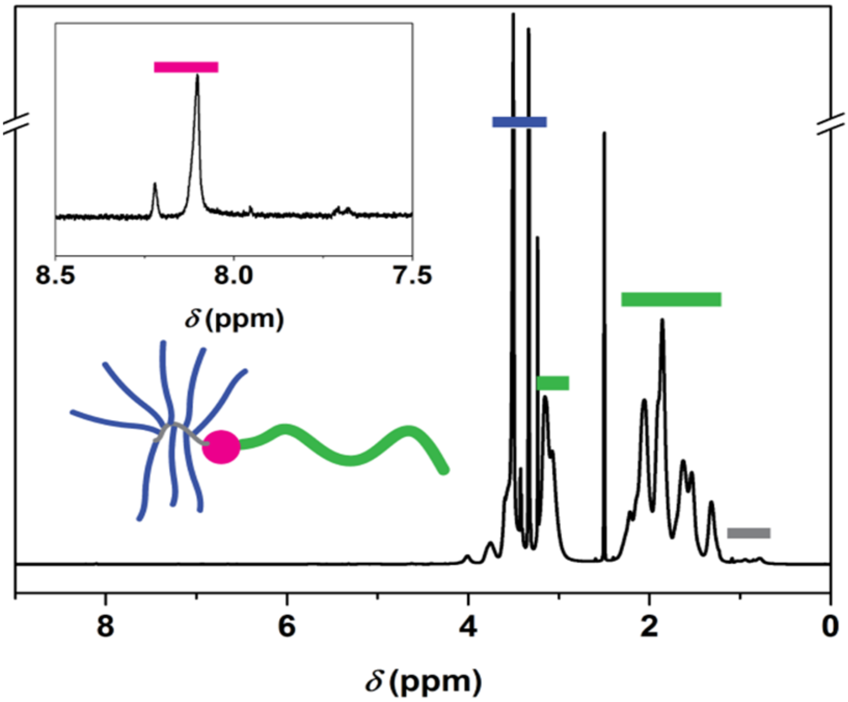

Figure S7. A representative ^{1}H-NMR spectrum of the corresponding PVP_{30k}-*b*-$POEGMA_{13k}$ block copolymer recorded at 400 MHz in DMSO-d_6 (signals from PVP in green, signals from POEGMA in blue, signals from the backbone in grey, signals from the triazole in pink).

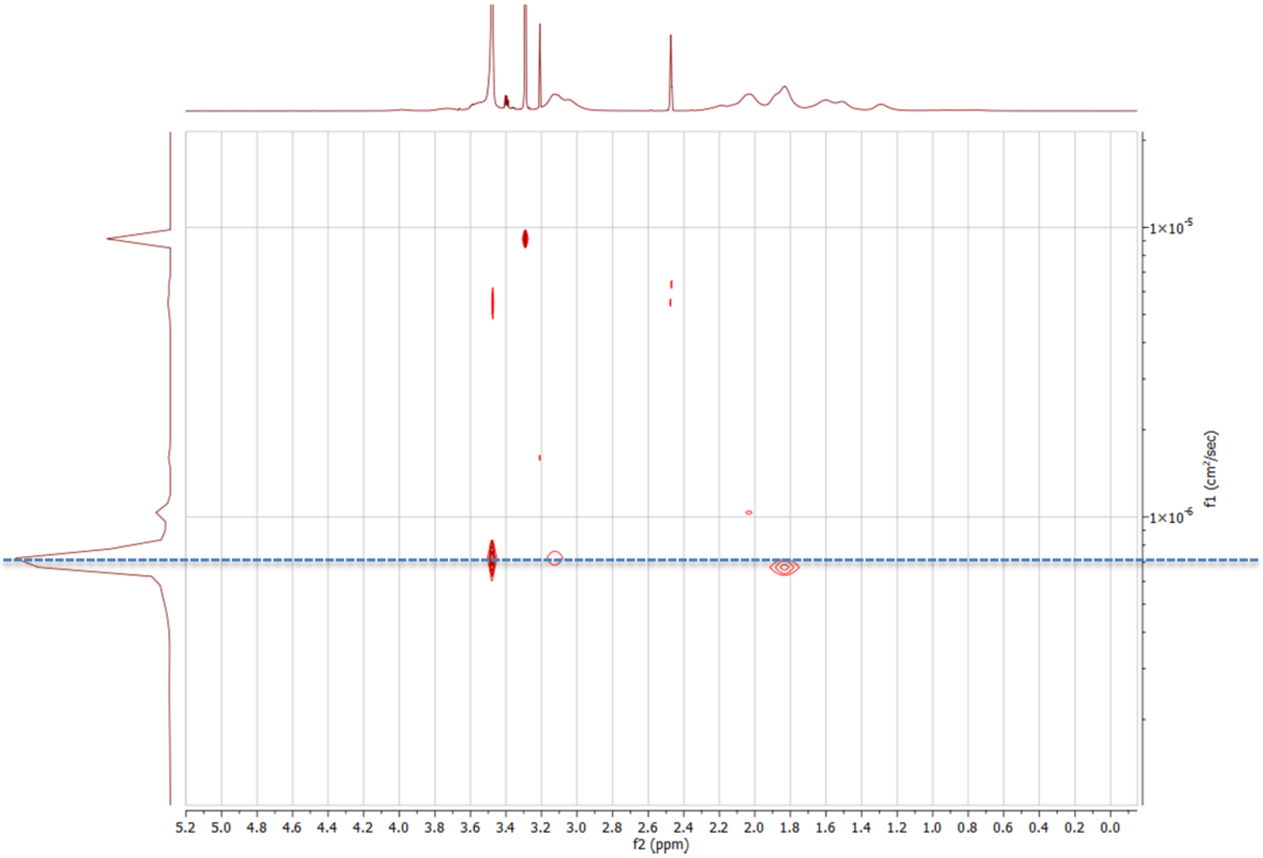

Figure S8. A representative DOSY spectrum of the corresponding PVP_{30k}-*b*-$POEGMA_{13k}$ block copolymer recorded at 600 MHz in DMSO-d_6 (the line denotes the diffusion coefficient of the block copolymer signals).

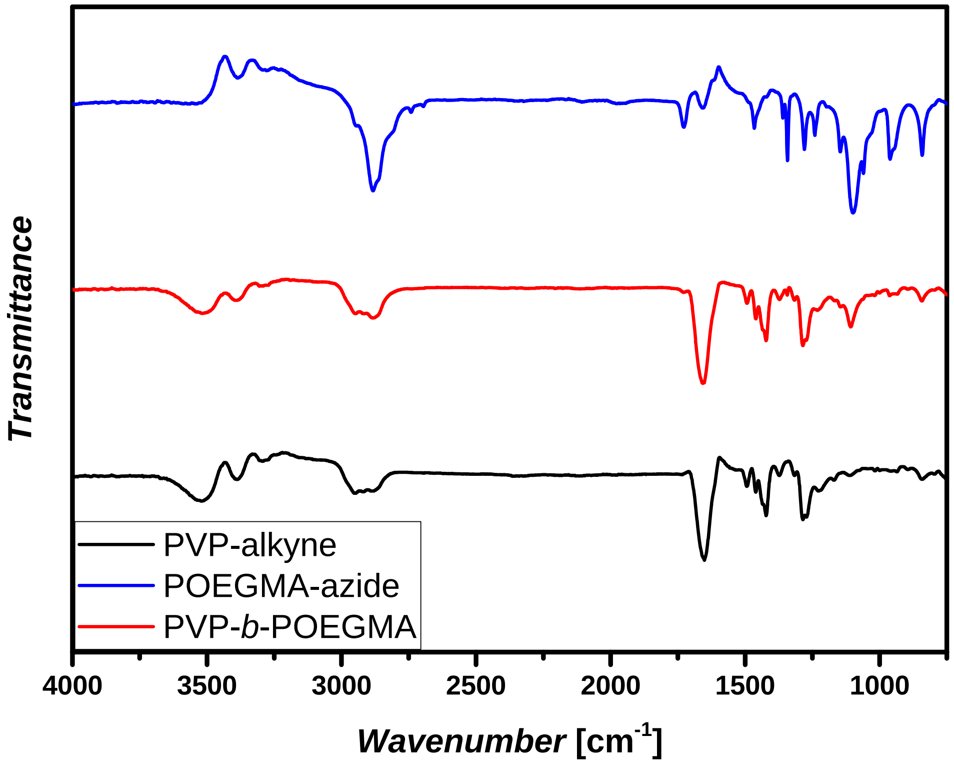

Figure S9. FT-IR spectra of $POEGMA_{13k}$, PVP_{24k} and PVP_{24k}-*b*-$POEGMA_{13k}$ recorded at 25°C.

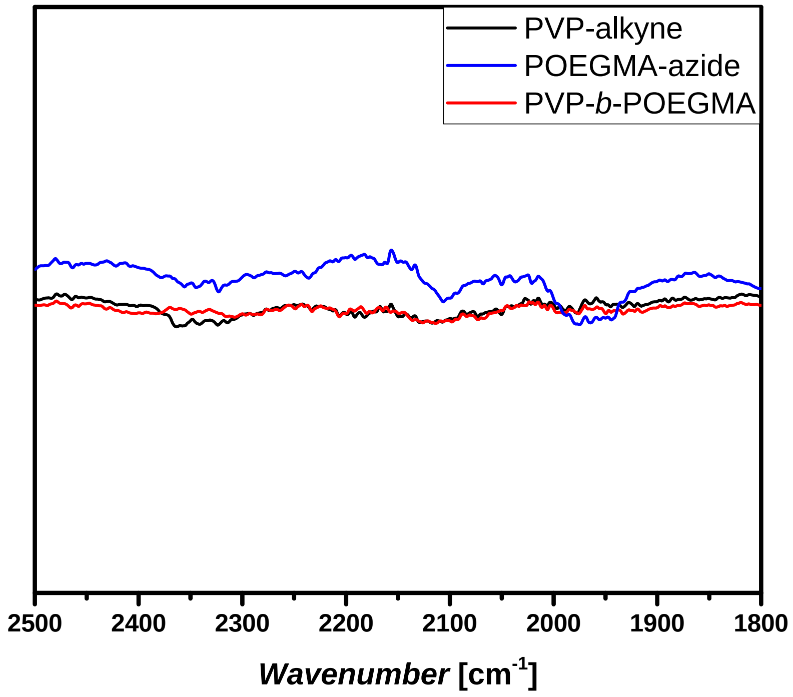

Figure S10. Magnification of FT-IR spectra of $POEGMA_{13k}$, PVP_{24k} and PVP_{24k}-*b*-$POEGMA_{13k}$ recorded at 25 °C.

Table S3. DLS results of PVP_{24k}-*b*-$POEGMA_{13k}$ before and after the addition of α-CD illustrating the efficiency of the self-assembly by comparing the ratio between the abundance of the unimer fraction (1st peak) and aggregate fraction (2nd peak).

Entry #	***DHBC conc.* (wt%)**	***Amount of α-CD (wt%)***	**R_{app} 1st peak (nm)**	**R_{app} 2nd peak (nm)**	**Normalized abundance ratio 1st peak : 2nd peak**
1	2.0	4.0	86.4	463.4	0.12 : 1
2	2.0	3.0	16.5	374.0	0.04 : 1
3	2.0	2.0	8.0	373.2	0.06 : 1
4	2.0	1.0	5.0	293.5	0.18 : 1
5	2.0	0	5.0	229.8	0.21 : 1
6	0.5	1.0	6.2	226.0	0.10 : 1
7	0.5	0.75	5.0	182.2	0.12 : 1
8	0.5	0.5	5.0	142.9	0.23 : 1
9	0.5	0.25	6.3	181.7	0.36 : 1
10	0.5	0	6.3	112.2	0.58 : 1
11	0.1	0.2	4.9	140.0	0.16 : 1
12	0.1	0.15	5.0	143.4	0.30 : 1
13	0.1	0.1	6.3	112.4	0.51 : 1
14	0.1	0.05	6.3	88.4	0.67 : 1
15	0.1	0	6.3	181.5	0.82 : 1

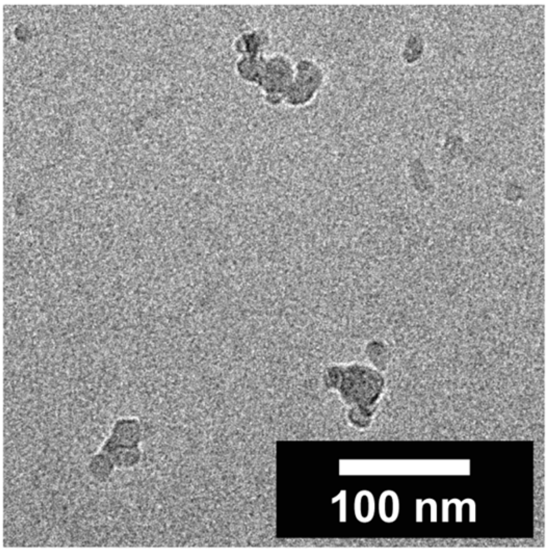

Figure S11. Additional cryo-TEM image of PVP$_{24k}$-*b*-POEGMA$_{13k}$ at a concentration of 1.0 wt%.

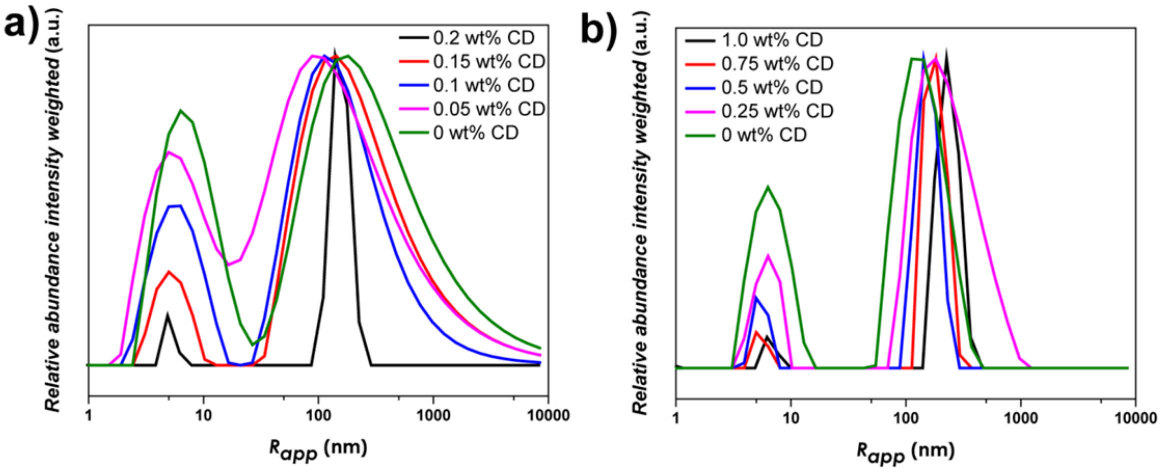

Figure S12. a) Intensity weighted particle size distribution of 0.1 wt% PVP$_{24k}$-*b*-POEGMA$_{13k}$ in Millipore water and the addition of α-CD (0.2 wt%, 0.15 wt%, 0.1 wt%, 0.05 wt% and 0 wt%) measured via DLS at 25 °C; b) Intensity weighted particle size distribution of 0.5 wt% PVP$_{24k}$-*b*-POEGMA$_{13k}$ in Millipore water and the addition of α-CD (1.0 wt%, 0.75 wt%, 0.5 wt%, 0.25 wt% and 0 wt%) measured via DLS at 25 °C.

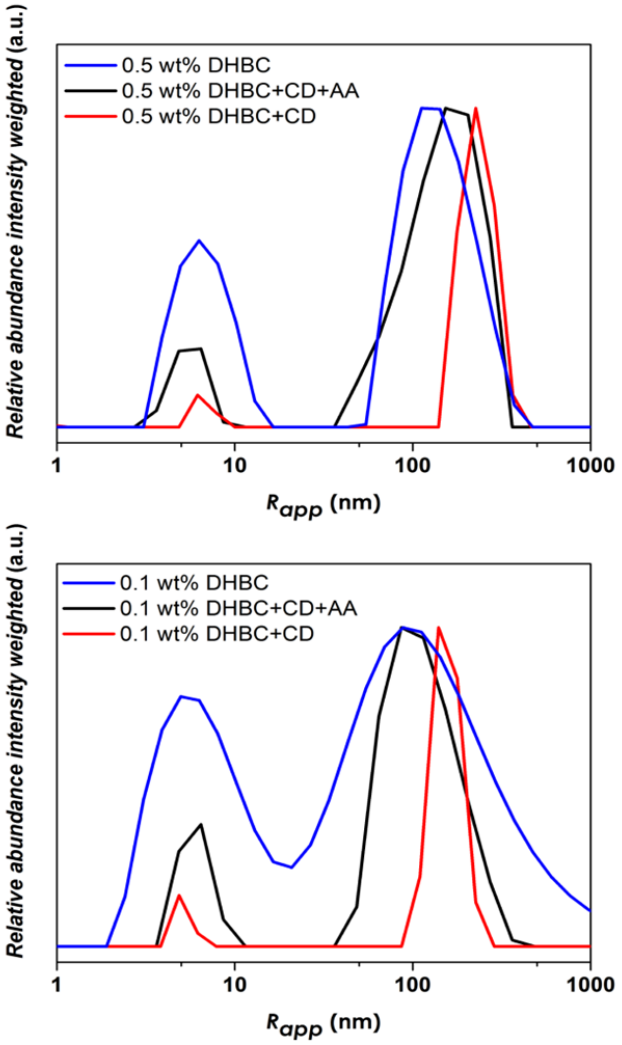

Figure S13. DLS results of PVP_{24k}-*b*-$POEGMA_{13k}$ before crosslinking, after crosslinking and after the addition of AA.

References

1. Quemener, D.; Davis, T. P.; Barner-Kowollik, C.; Stenzel, M. H., *Chem. Commun.* **2006,** (48), 5051-5053.

2. Willersinn, J.; Bogomolova, A.; Brunet Cabré, M.; Schmidt, B. V. K. J., *Polym. Chem.* **2017,** *8*, 1244-1254.
